# 268. Dengue - SARS-CoV-2 co-infection: Experience in hospitalized patients during COVID-19 pandemic

**DOI:** 10.1093/ofid/ofac492.346

**Published:** 2022-12-15

**Authors:** Fernando Rosso, Isabel L Zapata-Vasquez, Sarita Rodriguez, Diana M Martinez, Olga Agudelo, Sebastián Robles-Castillo, Alejandro Tejada

**Affiliations:** Fundación Valle del Lili, Cali, Valle del Cauca, Colombia; Fundacion Valle del Lili, Cali, Valle del Cauca, Colombia; Fundación Valle del Lili, Cali, Valle del Cauca, Colombia; Fundacion Valle del Lili, Cali, Valle del Cauca, Colombia; Fundacion Valle del Lili, Cali, Valle del Cauca, Colombia; Universidad Icesi, Cali, Valle del Cauca, Colombia; Universidad Icesi, Cali, Valle del Cauca, Colombia

## Abstract

**Background:**

Dengue fever and COVID-19 co-infection constitute a significant public health concern in Latin America, becoming a clinical challenge to distinguish these two entities in early stages of the disease. Clinical outcomes of coinfected hospitalized patients have not been well established.

**Methods:**

A cross-sectional study was conducted. We included suspected patients diagnosed with COVID-19/dengue co-infection admitted at Hospital Fundación Valle del Lili, Cali – Colombia, from March 2020 to March 2021. All dengue patients had positive NS1 and/or IgM dengue antibodies. SARS-CoV-2 infection was confirmed by RT-PCR or antigen rapid test from nasopharyngeal swab. Laboratory and clinical data were recollected from the clinical laboratory database, clinical charts, and institutional COVID-19 registry.

**Results:**

A total of 90 COVID-19 patients were included. 72 patients were confirmed only with COVID-19, and 18 with dengue co-infection. Most patients were male: 46 (63.9%) vs. 13 (72.2%). None of these study patients were vaccinated against COVID-19 or dengue. The median time from symptoms onset and the diagnosis was five days, and fever was the most common symptom for both groups. There were significant differences between COVID-19 patients and coinfected patients regarding presence of dyspnea (22.2% vs. 61.1%; p=0.003), desaturation (13.4% vs. 53.3%; p=0.002) and a higher neutrophil/lymphocyte ratio (NLR) (3.84 vs. 5.59; p = 0.038). The co-infection was associated with a worse presentation of the COVID-19 infection (p=0.002), an increased requirement of initial supplemental oxygen therapy (p=0.007), mechanical ventilation (p=0.0004), ICU management at the admission (p=0.002), and ICU final management (p=0.002). Overall mortality in patients with co-infection was 44.4% vs. 6.9% in only COVID-19 infected patients (p< 0.001).

**Conclusion:**

Despite the pandemic era, the possibility of co-infection of these two entities must be considered. Admitted coinfected patients were associated with worse clinical outcomes and higher mortality. According to our results, patients with co-infection present with severe respiratory symptoms and an elevated NLR. The impact of the Covid 19 vaccination on this coinfection is unknown.

**Disclosures:**

**Olga Agudelo, n/a**, Fundación valle del lili: Board Member.

